# Risk factors and predictive model for cervical lymph node metastasis in papillary thyroid carcinoma of the isthmus: a retrospective analysis of clinical and ultrasonographic features

**DOI:** 10.3389/fonc.2026.1794573

**Published:** 2026-05-04

**Authors:** Jia-Sheng Ding, Min Zhang, Fangfang Zhou

**Affiliations:** 1 Department of Intensive Care Unit, Lishui Central Hospital, The Fifth Affiliated Hospital of Wenzhou Medical University, Lishui, Zhejiang, China; 2 Department of Pathology, Lishui Central Hospital, The Fifth Affiliated Hospital of Wenzhou Medical University, Lishui, Zhejiang, China; 3 Department of Ultrasound, Lishui Central Hospital, The Fifth Affiliated Hospital of Wenzhou Medical University, Lishui, Zhejiang, China

**Keywords:** isthmus, lymph node metastasis, papillary thyroid carcinoma, predictive model, risk factors, ultrasonography

## Abstract

**Objective:**

This study aimed to investigate the clinical and ultrasonographic characteristics of papillary thyroid carcinoma (PTC) located in the thyroid isthmus and to identify independent risk factors predicting cervical lymph node metastasis (LNM).

**Methods:**

We retrospectively analyzed 445 patients with pathology-confirmed isthmic PTC who underwent surgery at Lishui Central Hospital between December 2020 and August 2025. Based on postoperative histopathology, patients were categorized into LNM (n=160) and non-LNM (n=285) groups. Clinical and preoperative ultrasonographic features were compared between groups. Univariate and multivariate logistic regression analyzes were performed to identify independent risk factors for LNM. A predictive model was constructed and evaluated using receiver operating characteristic (ROC) curve analysis.

**Results:**

Compared to the non-LNM group, patients in the LNM group were significantly younger (median age: 44.0 vs. 49.0 years, P = 0.012), had a higher proportion of females (70.0% vs. 62.1%, P = 0.018), and presented with larger tumors (median diameter: 9.5 vs. 6.0 mm, P < 0.001). Multivariate analysis identified maximum tumor diameter (OR = 1.15, 95% CI: 1.09–1.22, P < 0.001), female gender (OR = 1.82, 95% CI: 1.20–2.76, P = 0.004), and the presence of microcalcifications on ultrasound (OR = 1.58, 95% CI: 1.02–2.45, P = 0.040) as independent risk factors for LNM. The predictive model integrating these three factors yielded an area under the ROC curve (AUC) of 0.799 (95% CI: 0.762–0.837), with a sensitivity of 81.2% and a specificity of 66.7% at the optimal cutoff.

**Conclusion:**

Tumor size, female gender, and the presence of microcalcifications are independent preoperative risk factors for cervical LNM in isthmic PTC. A model based on these clinically accessible parameters provides a practical tool for preoperative risk assessment, which could help guide more individualized surgical management.

## 1 Introduction

Thyroid carcinoma is the most prevalent malignancy of the head and neck region, with its global incidence exhibiting a consistent upward trend in recent years (1). Papillary thyroid carcinoma (PTC) is the predominant histological subtype, accounting for approximately 90% of all thyroid cancers. A significant clinical characteristic of PTC is its propensity for early cervical lymph node metastasis (LNM), which is present in a substantial proportion of patients at diagnosis. The presence of LNM necessitates a shift from active surveillance to surgical intervention and critically influences the extent of surgery. Furthermore, LNM is a well-established risk factor for local recurrence, distant metastasis, and reduced survival rates (2).

Emerging evidence suggests that tumor location is a significant determinant of metastatic behavior. Specifically, PTC originating from the thyroid isthmus appears to harbor a higher risk of LNM compared to tumors located in the lateral lobes (3, 4), which may be related to its unique anatomical features (5).

Ultrasonography (US) serves as the primary, non-invasive imaging modality for the initial evaluation of thyroid nodules and regional lymph nodes due to its accessibility, real-time capability, and repeatability (6). However, a significant diagnostic challenge persists in the preoperative assessment of central compartment LNM. The deep-seated location of central lymph nodes often limits the sensitivity of conventional US, with reported detection rates as low as 30%, leading to potential under-staging (7).

Given the clinical implications of LNM and the diagnostic limitations in assessing the central compartment, identifying reliable preoperative predictors for metastasis in isthmic PTC is of paramount importance. While several studies have investigated risk factors for LNM in general PTC populations, data specifically focused on the isthmic subset remain relatively scarce (3, 8, 9). Clarifying the association between specific sonographic features of the primary tumor and the likelihood of LNM could enhance risk stratification, inform surgical planning, and optimize patient management.

Therefore, this study aimed to investigate the ultrasonographic characteristics and identify independent risk factors associated with cervical LNM in patients with papillary thyroid carcinoma located in the isthmus. The findings are expected to contribute to a more nuanced preoperative evaluation and support clinical decision-making for this distinct patient subgroup.

## 2 Materials and methods

### 2.1 Patients

**Figure 1:**
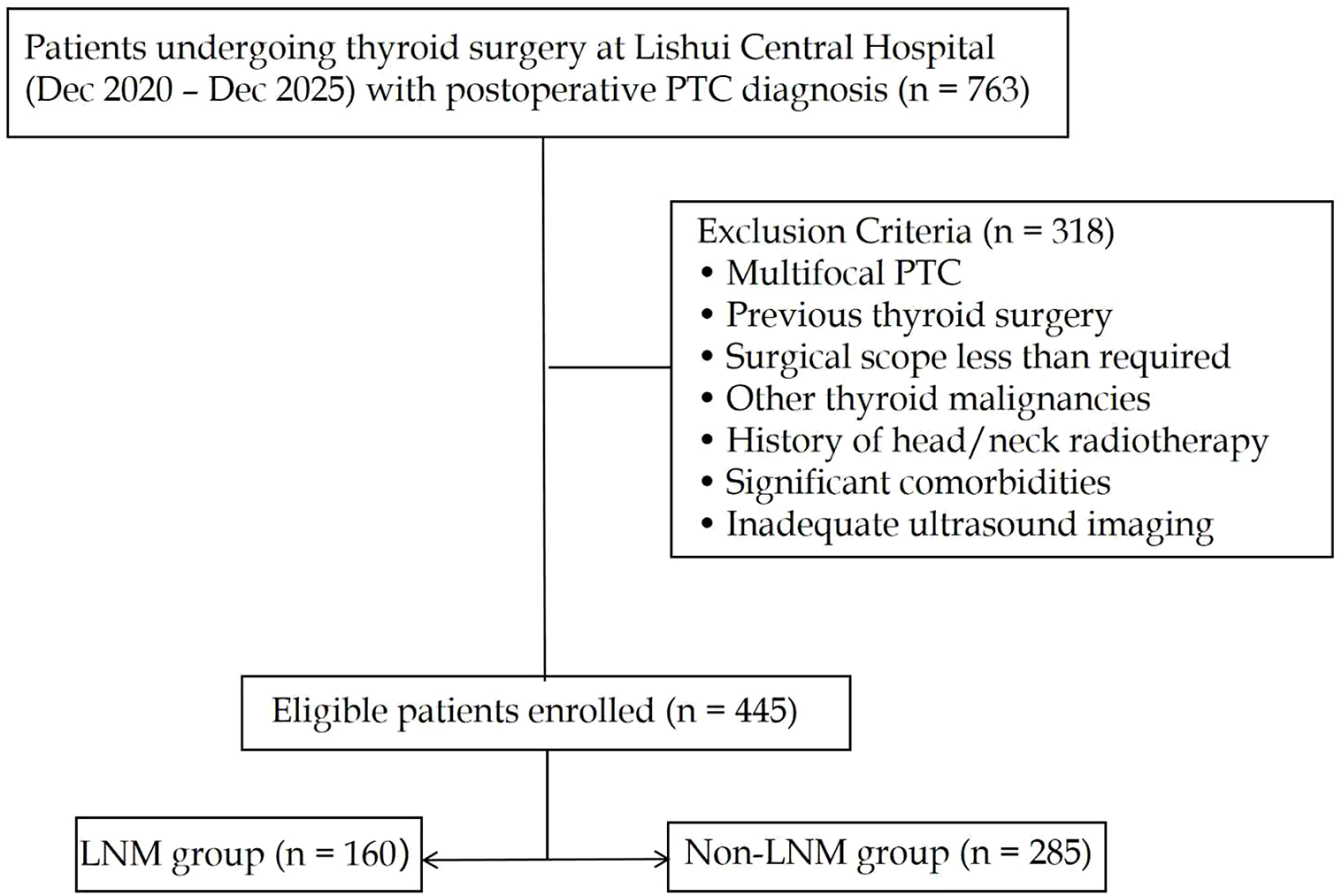
Flowchart of patient selection. (PTC, Papillary thyroid carcinoma; LNM, Lymph node metastasis).

This retrospective study included patients who underwent thyroid surgery at Lishui Central Hospital between December 2020 and August 2025. Initially, 763 patients with postoperative pathological confirmation of PTC were identified. The inclusion criteria were: 1) a solitary nodule located in the thyroid isthmus, confirmed by preoperative ultrasound and postoperative pathology; 2) surgical treatment comprising at least lobectomy with ipsilateral central lymph node dissection; 3) complete clinical, ultrasonographic, and pathological records. All patients underwent at least ipsilateral central neck dissection (CND); for tumors located in the mid-isthmus or with preoperative suspicion of bilateral metastasis, bilateral CND was performed. The extent of dissection was determined by the attending surgeon based on preoperative imaging and intraoperative findings. For the purpose of this study, only the presence or absence of pathologically confirmed LNM (regardless of laterality) was analyzed. Exclusion criteria included multifocal PTC, previous thyroid surgery, surgical scope less than required, other thyroid malignancies, history of head/neck radiotherapy, significant comorbidities, and inadequate ultrasound imaging. After exclusions, 445 patients with isthmic PTC were enrolled and categorized based on pathological lymph node status into metastasis (LNM) and non-metastasis (non-LNM) groups (**Figure 1**).

This study was approved by the Institutional Review Board of Lishui Central Hospital, and the requirement for informed consent was waived due to the retrospective design.

### 2.2 Measurements

Demographic, serological, and pathological data were extracted from the medical records. Preoperative thyroid ultrasonography was performed by experienced sonologists utilizing high-resolution color Doppler systems (Hitachi Arietta 70, Samsung Medison RS80A, Mindray R9S) equipped with linear array transducers (ML6-15, 14L5, L14-3WU) operating at 9–15 MHz, with imaging parameters individually optimized for each patient. With the patient in a supine position and the neck hyperextended, a comprehensive evaluation of the isthmic nodule was conducted via multi-planar two-dimensional grayscale ultrasound, color Doppler flow imaging (CDFI), and elastography when available. The thyroid isthmus was defined as the parenchyma anterior to the trachea connecting the bilateral lobes. On the maximal transverse section, vertical lines along the lateral tracheal edges demarcated the isthmus from the lobes; the isthmus was then trisected along this axis to classify nodule location as mid-isthmic (central third), left-sided isthmic (left third), or right-sided isthmic (right third). Assessed sonographic features and their definitions were as follows: maximum tumor diameter was recorded; composition was categorized as cystic/almost cystic, spongiform, mixed cystic-solid, or almost completely solid; margins were classified as smooth, ill-defined, lobulated/irregular, or exhibiting extrathyroidal extension; echogenicity was described as hypoechoic (relative to thyroid parenchyma), isoechoic, hyperechoic, markedly hypoechoic (relative to strap muscles), or anechoic; calcifications were classified as none, microcalcifications (defined as non-shadowing punctate hyperechoic foci ≤1 mm without comet-tail artifacts, which helped distinguish them from colloid crystals that typically exhibit comet-tail artifacts), macrocalcifications (coarse plaques >1 mm), or rim calcifications; the aspect ratio (taller-than-wide) was determined from the ratio of the maximum anteroposterior to transverse diameter (≥1 considered positive); vascularity was semi-quantitatively graded via the Adler scale (0: none; I: minimal, 1–2 dot/short linear signals; II: moderate, 3–4 dots or one main vessel with branches; III: marked, multiple vessels forming a network); and elastography, when performed, employed a 5-point color score (1: entirely/mostly green; 2: predominantly green with peripheral blue; 3: mixed blue-green, green predominating; 4: mixed blue-green, blue predominating; 5: nodule and surrounding tissue predominantly blue).

### 2.3 Statistical methods

Statistical analyzes were performed using SPSS version 25.0 (IBM Corp). Continuous variables were described as mean ± standard deviation or median (interquartile range) and compared using the independent t-test or Mann-Whitney U test, as appropriate. Categorical variables were expressed as frequencies (percentages) and compared using the Chi-square test or Fisher’s exact test. Univariate logistic regression was performed to identify variables associated with LNM. Variables with P < 0.05 in univariate analysis were entered into a multivariate binary logistic regression model (forward stepwise method) to identify independent risk factors. We acknowledge the limitations of stepwise regression, but our adequate sample size and cross-validation results support the robustness of the final model. Results are presented as odds ratios (OR) with 95% confidence intervals (CI). A predictive model was constructed using the independent risk factors and evaluated via receiver operating characteristic (ROC) curve analysis. The area under the curve (AUC), sensitivity, and specificity at the optimal cutoff were calculated. A two-sided P-value < 0.05 was considered statistically significant. To assess the robustness of the predictive model and to guard against overfitting, internal validation was performed using 10-fold cross-validation. The dataset was randomly partitioned into 10 equal-sized subsets; in each iteration, 9 subsets were used for training and the remaining 1 subset for validation. The process was repeated 10 times, and the average area under the ROC curve (AUC) was calculated across all folds.

## 3 Results

### 3.1 Patient characteristics

A total of 445 patients with isthmic PTC were included. Based on postoperative pathology, 160 patients (36.0%) were classified into the LNM group and 285 patients (64.0%) into the non-LNM group.

### 3.2 Comparison of baseline clinical and pathological features

The baseline characteristics of the two groups are summarized in **Table 1**. Significant differences were observed in age, gender, and maximum tumor diameter between the LNM and non-LNM groups (all P<0.05), Patients in the LNM group were significantly younger than those in the non-LNM group (median age: 44.0 vs. 49.0 years, P = 0.012), more likely to be female (70.0% vs. 62.1%, P = 0.018), and had larger tumors (median diameter: 9.5 mm vs. 6.0 mm, P<0.001). No significant differences were found in preoperative TSH, thyroglobulin, TPOAb levels, or tumor location within the isthmus(all P>0.05).

**Table 1:** Comparison of baseline characteristics between LNM and non-LNM groups.

Characteristic	Non-LNM (n=285)	LNM (n=160)	Statistical test	P-value
Age (years), median (IQR)	49.0 (38.0–57.0)	44.0 (34.0–54.0)	Mann-Whitney U	0.012
Female, n (%)	177 (62.1%)	112 (70.0%)	Chi-square	0.018
Tumor size (mm), median (IQR)	6.0 (4.0–8.0)	9.5 (7.0–13.0)	Mann-Whitney U	<0.001
TSH (mU/L), median (IQR)	1.58 (1.10–2.40)	1.60 (1.12–2.35)	Mann-Whitney U	0.842
Location in isthmus, n (%):			Chi-square	0.256
– Mid	32 (11.2%)	24 (15.0%)		
– Left	120 (42.1%)	66 (41.2%)		
– Right	133 (46.7%)	70 (43.8%)		

### 3.3 Comparison of ultrasonographic features

Significant differences were observed in margin, aspect ratio, calcification type, and CDFI grade between the two groups (all <0.05), as shown in **Table 2**. The LNM group exhibited a higher proportion of irregular/extrathyroidal extension margins (65.0% vs. 45.3%), microcalcifications (38.1% vs. 24.6%), and higher CDFI grades (Grade II–III: 15.6% vs. 8.1%). No significant differences were found in composition, echogenicity, or elasticity score (all >0.05).

**Table 2:** Comparison of ultrasonographic features between LNM and non-LNM groups.

Feature	Non-LNM (n=285)	LNM (n=160)	P-value
Margin, n (%)			<0.001
– Smooth	0 (0%)	0 (0%)	
– Ill-defined	95 (33.3%)	32 (20.0%)	
–Lobulated/Irregular	61 (21.4%)	24 (15.0%)	
–Extrathyroidal extension	129 (45.3%)	104 (65.0%)	
Aspect ratio ≥1, n (%)	98 (34.4%)	40 (25.0%)	0.042
Calcification, n (%)			0.003
– None	158 (55.4%)	72 (45.0%)	
– Macrocalcification	35 (12.3%)	18 (11.2%)	
– Rim calcification	2 (0.7%)	1 (0.6%)	
– Microcalcification	70 (24.6%)	61 (38.1%)	
CDFI Grade II–III, n (%)	23 (8.1%)	25 (15.6%)	0.015

### 3.4 Multivariate logistic regression analysis for LNM

**Figure 2:**
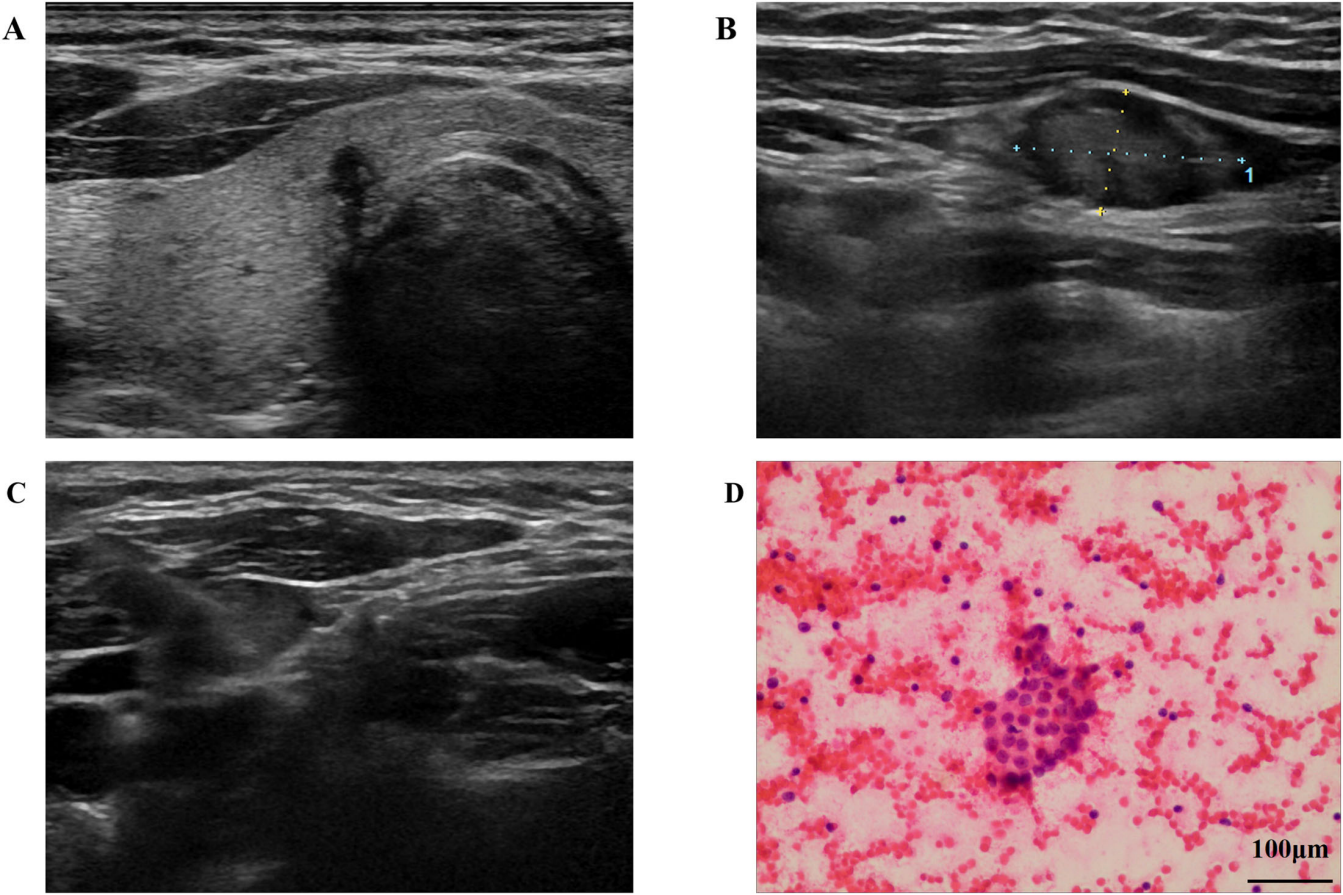
Ultrasound and cytopathological findings in a 36-year-old female patient with papillary thyroid carcinoma originating from the thyroid isthmus. **(A)** Transverse grayscale ultrasound image demonstrates a hypoechoic nodule (white arrow) in the left portion of the thyroid isthmus, containing multiple hyperechoic foci. **(B)** Grayscale ultrasound of the left neck reveals a suspicious lymph node (white arrow) at level IV. **(C)** Ultrasound-guided fine-needle aspiration (FNA) of the suspicious lymph node. **(D)** Cytopathology from the FNA specimen confirms metastatic carcinoma consistent with PTC (Papanicolaou stain, ×200).

Variables with P<0.05 in univariate analyzes (age, gender, tumor size, margin, aspect ratio, microcalcification, CDFI grade) were included in the multivariate model. The results identified maximum tumor diameter (OR = 1.15, 95%CI: 1.09–1.22), female gender (OR = 1.82, 95%CI: 1.20–2.76), and presence of microcalcification (OR = 1.58, 95%CI: 1.02–2.45) as independent risk factors for LNM in isthmic PTC (**Table 3**). **Figure 2** demonstrates representative ultrasound images of an isthmic papillary thyroid carcinoma, illustrating key sonographic features associated with the tumor.

**Table 3:** Multivariate logistic regression analysis for LNM in isthmic PTC.

Variable	β	Wald χ²	P-value	OR (95% CI)
Tumor size (mm)	0.14	22.5	<0.001	1.15 (1.09–1.22)
Female gender	0.60	8.1	0.004	1.82 (1.20–2.76)
Microcalcification	0.46	4.2	0.040	1.58 (1.02–2.45)
Constant	-2.10	25.3	<0.001	0.12

### 3.5 Diagnostic performance of the predictive model

**Figure 3:**
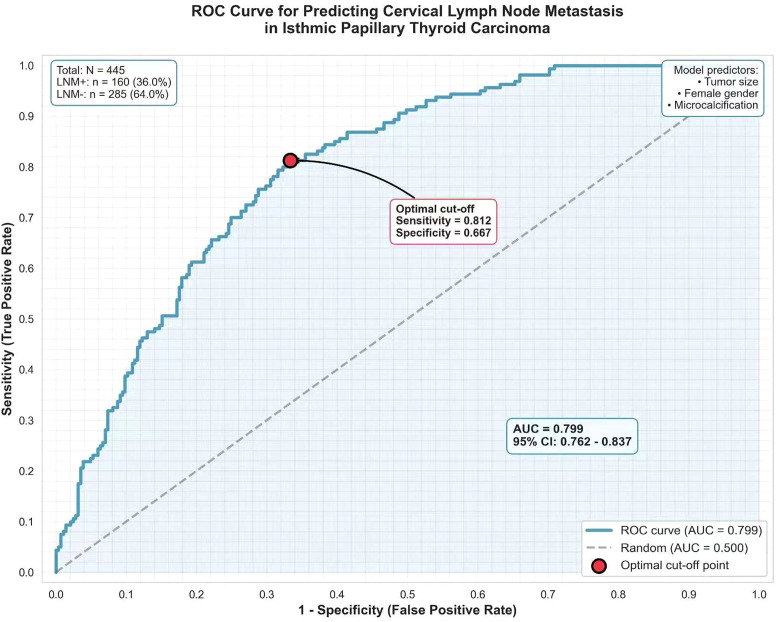
Receiver operating characteristic curve of the predictive model for lymph node metastasis in isthmic papillary thyroid carcinoma. The solid blue curve represents the performance of the prediction model, with an area under the curve (AUC) of 0.799(95% CI: 0.762–0.837). The dashed grey diagonal line indicates the reference line of a random classifier (AUC = 0.5). The red point on the ROC curve marks the optimal cut-off threshold, corresponding to a sensitivity of 81.2% and a specificity of 66.7%.

A model incorporating the three independent predictors (tumor size, female gender, microcalcification) was constructed. The ROC curve analysis yielded an AUC of 0.799 (95% CI: 0.762–0.837). At the optimal cutoff point, the sensitivity was 81.2% and specificity was 66.7%(**Figure 3**). The 10-fold cross-validation yielded a mean AUC of 0.782 (95% CI: 0.745–0.819), which is close to the original AUC, indicating minimal overfitting and acceptable model robustness.

### 3.6 Detailed characteristics of lymph node metastasis

Among the 160 patients with pathologically confirmed LNM, the detailed characteristics are summarized in **Table 4**. The majority of patients (71.9%) had metastasis confined to the central compartment. The median number of total excised lymph nodes was 8 (IQR: 5–10), of which a median of 2 (IQR: 1–3) were positive. The median diameter of the largest metastatic lymph node was 4.3 mm (IQR: 3.2–7.1).

**Table 4:** Detailed characteristics of patients with cervical lymph node metastasis (n=160).

Characteristic	Value
Central neck dissection (CND), n (%)	
– Ipsilateral CND only	384 (86.3%)
– Bilateral CND	61 (13.7%)
Lateral neck dissection (LND), n (%)	45 (10.1%)
Location of LNM, n (%)	
– Central compartment only	115 (71.9%)
– Lateral compartment only	16 (10.0%)
– Both central and lateral	29 (18.1%)
Number of excised lymph nodes per patient, median (IQR)	
– Total excised	8 (5–10)
– Positive (metastatic)	2 (1–3)
Diameter of the largest metastatic lymph node (mm), median (IQR)	4.3 (3.2–7.1)

## 4 Discussion

The unique anatomical milieu of the thyroid isthmus—including its rich lymphatic network and bilateral drainage pattern—likely underlies the distinct metastatic behavior of isthmic PTC. Compared with existing predictive models derived from mixed PTC populations, our model is specifically tailored to isthmic PTC and uses only three simple preoperative variables, offering practical advantages for routine use and complementing the 2025 ATA guidelines.

PTC arising from the thyroid isthmus, while less common than its lobar counterparts, demonstrates distinct clinical behavior that can be attributed to its unique anatomical milieu. Located centrally and anterior to the trachea, the isthmus exhibits a specialized vascular and lymphatic architecture. Key features include a higher density of lymphatic vessels, which may facilitate early invasion and create conduits for metastasis (10); a bilateral lymphatic drainage pattern that extends into the central compartments and superior mediastinum (level VII), predisposing to widespread nodal involvement (11); and a rich vascular supply with increased microvessel density that may further promote local invasion and dissemination (12). These collective anatomical characteristics likely underlie the greater propensity for capsular invasion and lymph node metastasis observed in isthmic PTC. In this context, the 36.0% LNM rate identified in our cohort aligns with and supports the premise of early, often bilateral lymphatic spread inherent to this region, thereby corroborating previous clinical observations that an isthmic tumor location confers an elevated metastatic risk compared to tumors originating in the thyroid lobes.

The identification of female sex as an independent risk factor presents a nuanced finding, contrasting with studies identifying male sex as a risk factor for larger-volume disease (13). Estrogen receptor signaling, particularly via GPER expressed in thyroid tissue, may promote lymphangiogenesis and facilitate nodal spread (14). Sex-based differences in immune surveillance could also contribute to differential metastatic behavior (15). Importantly, this finding is specific to the isthmic PTC subgroup and may not generalize to all PTCs. Independent confirmation in other isthmic PTC cohorts is needed. While surveillance bias (more frequent ultrasound in females) may contribute, its effect is attenuated by the variable’s persistence after multivariate adjustment. This association underscores the need to incorporate sex-specific considerations into the preoperative assessment of isthmic PTC.

Tumor size represents a well-established predictor of lymph node metastasis (LNM) in papillary thyroid carcinoma (PTC) (16). The predictive value of tumor size likely reflects underlying tumor biology, as larger nodules may indicate greater proliferative activity and invasive potential. Mechanistically, increased tumor volume may facilitate the breach of the thyroid capsule and provide a greater likelihood of encountering and invading regional lymphatic channels, thereby elevating the risk of nodal dissemination (17). Our findings, which identify larger tumor diameter as a significant independent risk factor for LNM in isthmic PTC, are consistent with this established oncological principle.

Our analysis—specifically focused on isthmic PTC—identifies microcalcifications as an independent predictor of nodal involvement. This discrepancy may be attributable to the distinct anatomical context of the isthmus, where smaller tumor volume might render microcalcifications an earlier marker of malignant behavior. Indeed, several studies have established microcalcifications as an independent risk factor for LNM in PTC (18–20). This observation is further supported by a multi-center radiomics study of 660 PTC patients, which confirms microcalcification as an independent risk factor for lymph node metastasis (LNM) in multivariate analysis. Moreover, the integration of microcalcification into a hybrid radiomics model demonstrated strong predictive performance across multiple cohorts (AUCs 0.841–0.883) (21). These consistent findings across different studies reinforce the role of microcalcification as a significant imaging marker, particularly in the anatomically confined isthmus region, where it may serve as an early indicator of aggressive tumor behavior and LNM risk.

In contrast, other calcification patterns—such as coarse calcifications or peripheral rim calcifications—carry lower clinical significance in isthmic PTC. Coarse calcifications are often associated with benign degenerative or inflammatory processes, such as nodular goiter or thyroid adenoma, while rim calcifications may indicate cystic degeneration or chronic inflammation and are seldom linked to malignancy (22). Thus, the detection of microcalcifications in an isthmic nodule should raise clinical suspicion for both malignancy and potential LNM.

The clinical relevance of this predictive model lies in its potential to translate preoperative assessments into practical guidance for surgical planning. For patients classified as high-risk based on the triad of larger tumor size, microcalcifications, and female sex, a more proactive surgical approach may be warranted. Given the increased likelihood of nodal involvement in such cases, bilateral central neck dissection could be considered (23), and further preoperative imaging might be helpful to evaluate the lateral compartments.

In contrast, for low-risk patients—typically those with smaller tumors without microcalcifications—a more conservative resection, such as isthmusectomy or lobectomy with isthmusectomy, might be appropriate. This aligns with contemporary emphasis on thyroid-preserving strategies and may help reduce surgical-related morbidity (24, 25). The model may also assist in identifying patients who could benefit from preoperative molecular testing (e.g., for BRAF V600E). A positive test result may provide additional support for a more comprehensive surgical approach in select high-risk cases with indeterminate features. Overall, this risk-stratification tool offers a practical framework for tailoring the extent of surgery in isthmic PTC, seeking to balance oncologic control with functional preservation.

Several limitations of this study should be noted. Its single-center retrospective design may introduce selection bias, and external validation in prospective multi-center cohorts from diverse geographic regions, with consistent ultrasound protocols, is urgently needed before clinical application. Furthermore, the reliance on ultrasound—with its operator dependency and limited sensitivity for central compartment micrometastases—remains a constraint. Although all ultrasound examinations were performed by experienced sonologists using standardized protocols, inter-observer variability is an inherent limitation. Future studies could incorporate automated quantitative ultrasound features (e.g., radiomics) to reduce this variability. The current model is based on routinely available clinical and sonographic variables; future integration of molecular markers and advanced imaging features could further enhance its predictive accuracy. We also acknowledge that calibration curve and decision curve analyzes were not performed, as our primary aim was risk factor identification and simple risk classification. These metrics will be considered in future prospective validation studies.

## 5 Conclusion

In summary, tumor size, female sex, and microcalcifications are key preoperative determinants of LNM in isthmic PTC. A predictive model based on these factors provides a practical instrument for risk assessment. By facilitating more nuanced surgical planning, this model complements guideline recommendations and may contribute to optimizing the therapeutic approach for patients with carcinoma of the thyroid isthmus.

## Data Availability

The raw data supporting the conclusions of this article will be made available by the authors, without undue reservation.
